# Accuracy of bone resection in total knee arthroplasty using CT assisted-3D printed patient specific cutting guides

**DOI:** 10.1051/sicotj/2018032

**Published:** 2018-07-13

**Authors:** Ikram Nizam, Ashish V. Batra

**Affiliations:** Ozorthopaedics, 1356 High Street, Malvern, VIC 3144 Australia

**Keywords:** Patient specific instrumentation, 3D cutting guides, Total knee arthroplasty, Bone resection

## Abstract

*Introduction*: We conducted this study to determine if the pre-surgical patient specific instrumented planning based on Computed Tomography (CT) scans can accurately predict each of the femoral and tibial resections performed through 3D printed cutting guides. The technique helps in optimization of component positioning determined by accurate bone resection and hence overall alignment thereby reducing errors.

*Methods*: Prophecy evolution medial pivot patient specific instrumented knee replacement systems were used for end stage arthrosis in all consecutive cases over a period of 20 months by a single surgeon. All resections (4 femoral and 2 tibial) were measured using a vernier callipers intraoperatively. These respective measurements were then compared with the preoperative CT predicted bone resection surgical plan to determine margins of errors that were categorized into 7 groups (0 mm to ≥2.6 mm).

*Results*: A total of 3618 measurements (averaged to 1206) were performed in 201 knees (105 right and 96 left) in 188 patients (112 females and 76 males) with an average age of 67.72 years (44 to 90 years) and average BMI of 32.3 (25.1 to 42.3). 94% of all collected resection readings were below the error margin of ≤1.5 mm of which 90% showed resection error of ≤1 mm. Mean error of different resections were ≤0.60 mm (*P* ≤ 0.0001). In 24% of measurements there were no errors or deviations from the templated resection (0.0 mm).

*Conclusion*: The 3D printed cutting blocks with slots for jigs accurately predict bone resections in patient specific instrumentation total knee arthroplasty which would directly affect component positioning.

## Introduction

Calibrated alignment and accurate bone resections have been shown to improve component positioning leading to increased longevity and success of total knee arthroplasty [1]. More recently patient-specific instrumented (PSI) TKA has gained popularity as a preferred option [2]. Manufacturers claim this advancement significantly reduces operative time, is less invasive, easier to use, minimises the risk of error by providing precise measurements and reduce theatre turn over times [3,4]. By not requiring the use of intramedullary rods to determine alignment, patient specific instrumentation avoids violation of the intramedullary canal, potentially reducing the risk of intraoperative fat embolism, which have been reported to be between 46% and 65% [5–7]. Other reported advantages include less risk of peri & post-operative blood loss [8,9]. Patient specific instrumentation aims at improving bone resection accuracy through custom cutting blocks constructed using preoperative 3-Dimensional (3D) imaging [10,11]. The surgical plan in combination with the cutting guides determine the resection thickness, component size, femoral rotation, femoral and tibial component alignment. Several clinical studies have shown that patient specific instrumentation is safe, accurate and reproducible in primary TKA. Accurate preparation of the femoral and tibial surfaces will determine component positioning and hence alignment/rotation and this in turn reflects on function and longevity [12,13].

## Materials and methods

The study was conducted prospectively with patients admitted between May 2016 and December 2017 in our institution. Patients with primary or secondary osteoarthritis OA and inflammatory arthritis who were suitable to undergo patient-specific TKA were included in the study. Patients that underwent revision TKA were excluded from the study.

Informed consent from patients and ethics approval was received for this study. Once patients had been listed for patient-specific TKA, they underwent an alignment hip, knee and ankle CT scan and a long leg X-ray of the involved lower limb as our standard protocol. The radiological information was used to create a virtual model of the patient's knee and a detailed preoperative plan was done with ideal femoral and tibial component sizing including the respective bone cuts. Cartilage offsets were applied by the manufacturer up to 2 mm for both tibial and distal femur cartilage. CT scan doesn't demonstrate articular cartilage thickness and therefore the blocks won't account for cartilage hence cartilage has to be approximated. These cartilage offsets were standard norms for “Prophecy evolution protocol” when using CT.

**Figure 1:**
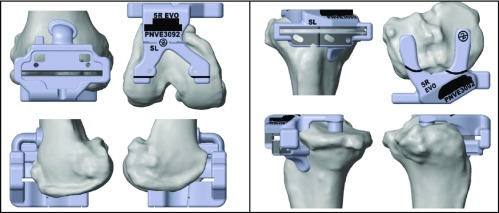
Femoral and tibial cutting block mounted on the distal femur and on the proximal tibia respectively. These are company manufactured patient specific femoral cutting blocks based on CT scan created virtual bone models. Multiple contact points on the femoral cutting block and the tibial cutting block, along with markings allow accurate positioning and rotational alignment improving accuracy of the cuts and femoral and tibial rotation respectively. There are slots for external jigs to double check slope and tibial base plate rotation.

**Figure 2:**
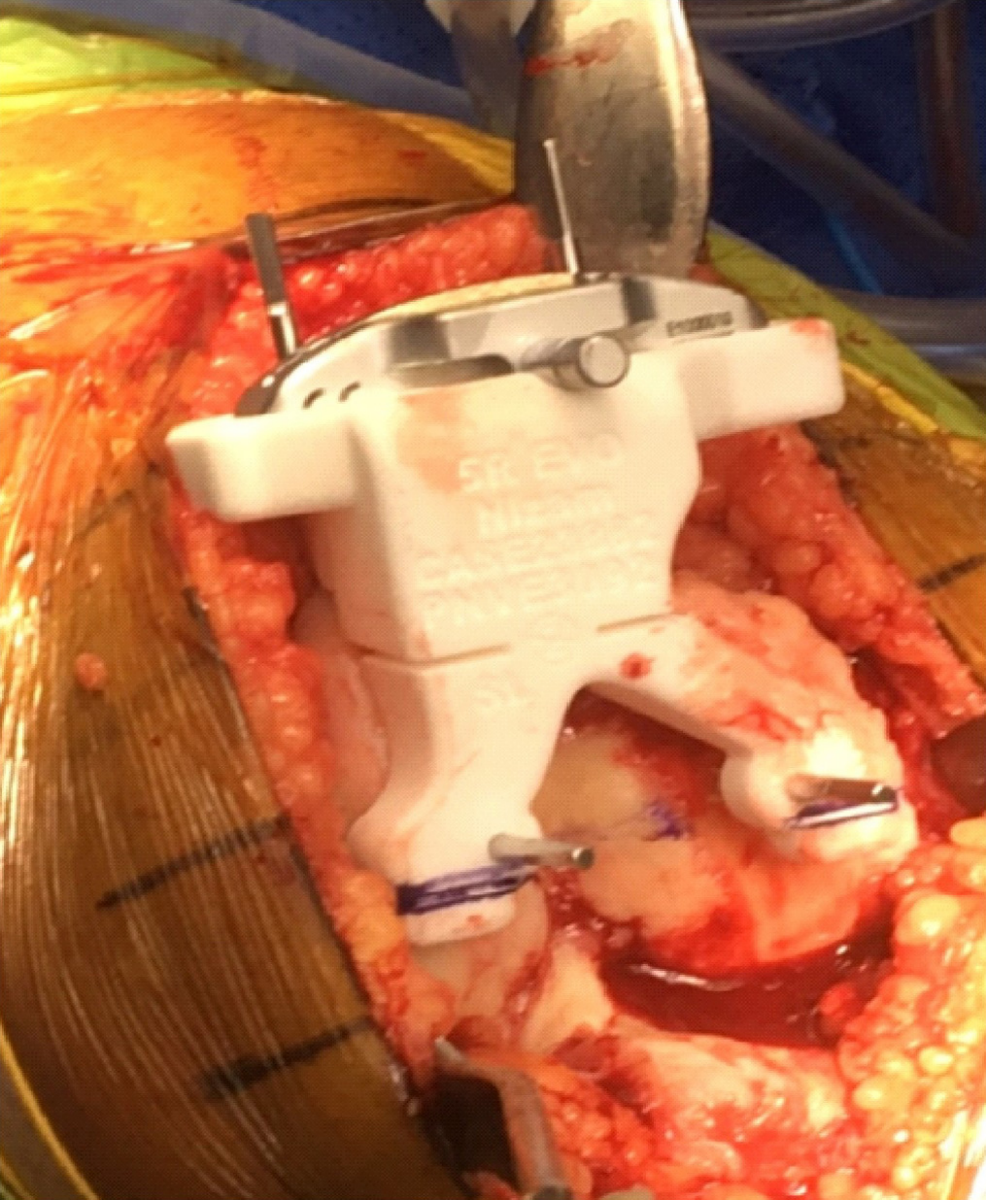
The PSI cutting block placed accurately on the femoral surface and pinned. An intraoperative image showing the patient specific cutting block placed accurately on the distal femoral surface − flexion and rotation checked. The block contains the cutting jig in situ. The block contains information related to the surgery.

**Figure 3:**
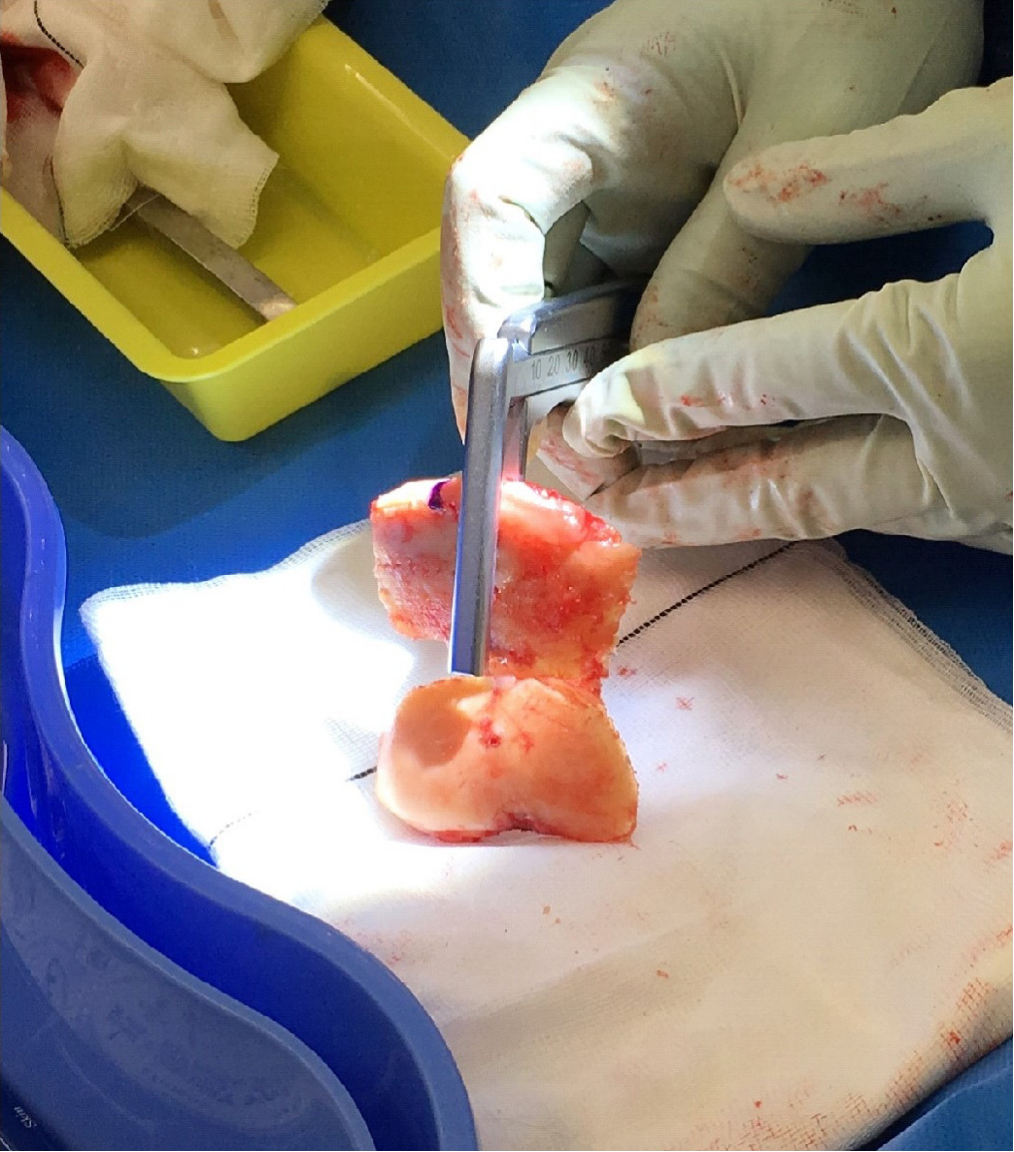
Measurement of the medial and lateral distal femoral condyle and posterior condylar cuts. Sequential pinning of the block was carried out very carefully with 2 distal pins (through the 3D block) followed by 2 anterior femoral pins (through the in situ jig). The oblique anterior femoral pin was placed last to prevent any movement of the 3D block whilst resecting. Distal femoral resections were then done through the in situ jig. The posterior condylar cuts were done through the appropriate standard 4:1 in block as per plan. The largest thickness of bony prominence was recorded by the senior surgeon using Vernier Calipers for each resected medial and lateral distal femoral condyle. Three separate measurements − horizontal, vertical and diagonal were made, and the average measurement was recorded independently.

The senior author designed markings on the femoral and tibial cutting blocks (Figures 1–3). Following surgeon approval of the surgical template and alignment of components in multiple planes, rapid-prototyping computer-assisted design and computer-assisted manufacturing technology were used to create the 3D patient specific cutting block. These blocks were printed and manufactured by PROPHECY^®^ (Arlington, TN, USA).

Evolution medial pivot patient-specific TKA system (Wright Medical, Surgical Specialties Pty Ltd) was used in all patients. Femoral preparation was done first. Once the PSI block was in place, it was pinned (Figure 2). The femoral block with the cutting jig in situ always sat accurately on the femoral surface, without any osteophytes being removed (Figure 2). The tibial block was seated as per the surgical guide and pinned (Figure 3). Local infiltration into the deep capsule and surrounding deep tissues was carried out that included a mixture of 250 to 300 mg of ropivacaine HCl (Naropin^®^, AstraZeneca Pty Ltd) 2.0 mg/ml, along with a standard 30 mg dose of ketorolac tromethamine (TORADOL^®^, Roche Products Pty Ltd), 10 μg/ml adrenaline and 2 g of tranexamic acid, as a part of our enhanced recovery programme [14,15].

Component sizing, ligament stability/balancing, range of movement and patella tracking were then performed with trial implants in all cases. The senior surgeon did not routinely resurface any patella but removed marginal osteophytes (after completion of resections) and circumferential denervation with diathermy. Both femoral and tibial components were cemented, and the appropriate poly liner inserted.

**Figure 4:**
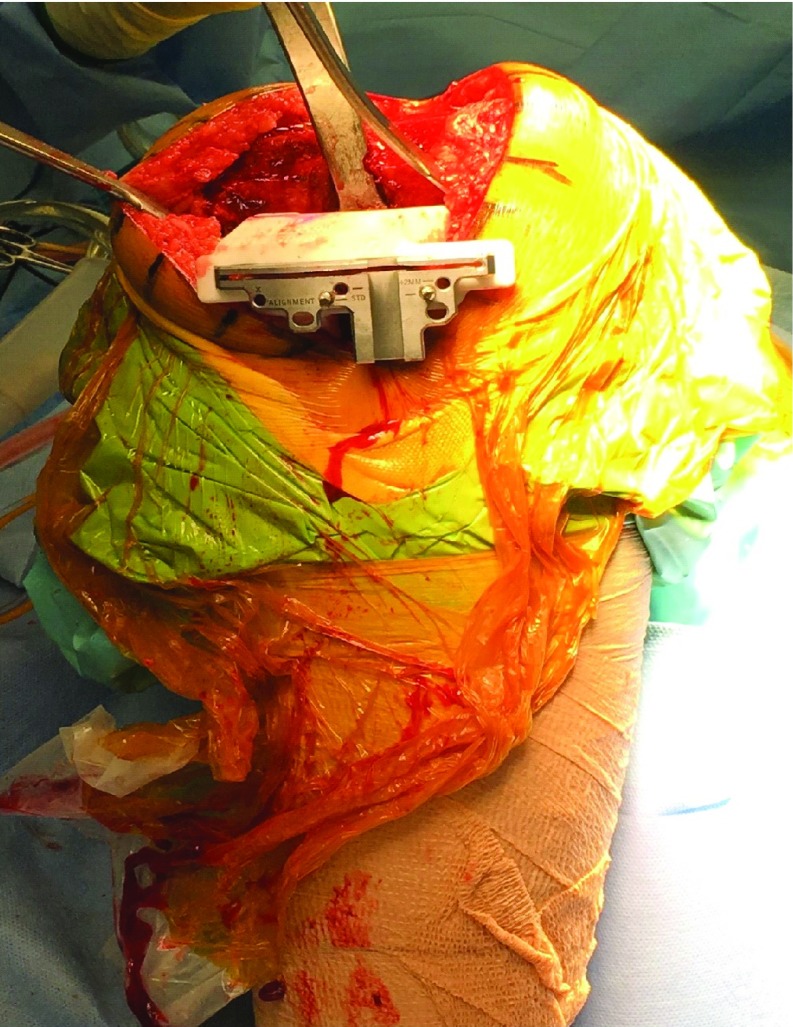
The tibial block was seated as per surgical plan and pinned. This is an intraoperative image showing the patient specific cutting block placed on the proximal tibial surface which perfectly sat, once soft tissue was dissected off the bony surfaces for the contact points of the block. Tibial rotation and slope was double checked by extra-medullary alignment guide through the 3D block and the oblique pin placed before the cuts were made.

**Figure 5:**
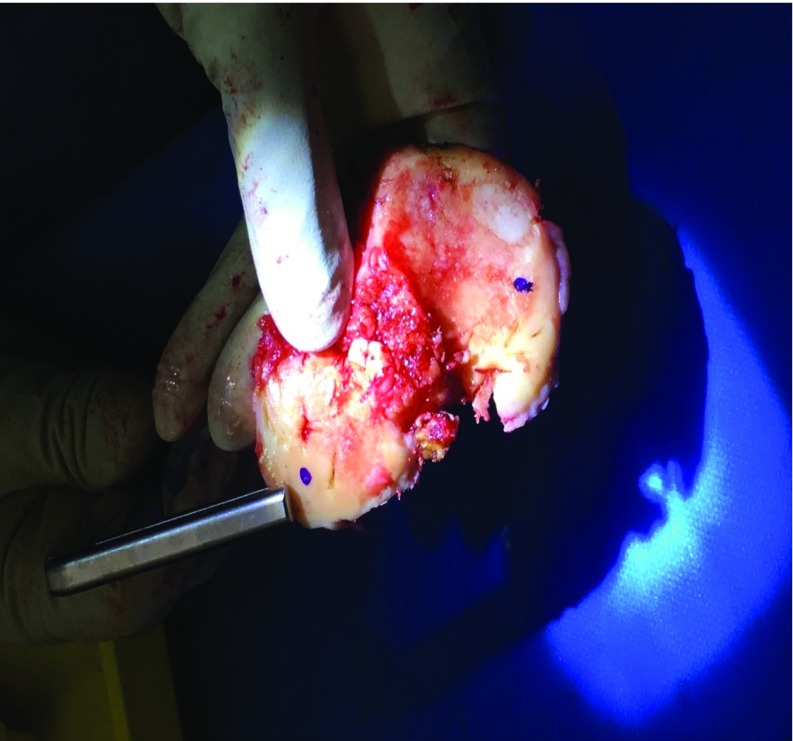
Tibial cut measured from the corresponding points of the plan and recorded for medial and lateral surfaces of tibial plateau. The cuts were measured by the senior surgeon using Vernier Calipers from the corresponding points on the plan (marked with a dot to ensure accuracy) and recorded for medial and lateral surfaces of tibial plateau. Three separate measurements were averaged per resection.

The largest thickness was recorded for all resections by averaging 3 separate measurements per resection − horizontal, vertical and diagonal (Figures 4 and 5). On the femoral side, distal medial condylar resection, distal lateral condylar resection, posterior medial condylar resection, and posterior lateral condylar resection were recorded. While on the tibial side, proximal medial plateau resection and proximal lateral plateau resection were noted.

The mean error between the actual and planned bone resections using the 3618 readings was derived from 201 knees. The accuracy of the intra-operative resection was the primary outcome of the study. A saw blade with the thickness of 1.27 mm was used for all TKAs. This was accounted for in the preoperative CT plans.

We calculated *P*-value using GraphPad Software. We derived these to account for extreme variations in data, if any.

## Results

201 PSI TKAs were included in our study of 188 patients. There were 118 females and 70 males with a mean age of 67.72 years (between 46 and 90 years). The average BMI was 32.3 (25.1 to 42.3). There were 105 right and 96 left-sided TKAs. Primary Osteoarthritis was the diagnosis in all patients, except 4 patients diagnosed with inflammatory arthritis.

The surgical time ranged from 46 to 102 min, with the mean operating time of 62 min from skin to skin. There were no major intra-operative complications or difficulties reported by the operating surgeon while using the cutting blocks.

A total of 3618 readings (averaged to 1206) were collected (18 per knee; 3 per resection, 4 femoral and 2 tibial resections) from 201 knees. Ninety-four percent (94%) of all collected readings were below the error margin of ≤1.5 mm of which 90% had error margins ≤1 mm compared to the original surgical plan (Tables 1 and 2). The highest error recorded was 6.7 mm for the posterior lateral femoral condyle (due to a mechanical problem in the standard 4 in 1 cutting jig sliding mechanism used for anterior, posterior and chamfer cuts). In 24% of measurements there were no errors or deviations from the templated resection (0.0 mm). The posterior condyle lateral resections recorded the highest mean error of all six resections at 0.60 mm (Table 3). The distal femoral condyle (DFC) medial resections had the lowest mean error (0.38 mm). There were no major differences in mean errors of different sizes of resection. They all ranged between 0.38 and 0.60 mm.

**Table 1:** Percentage of resection error. The table below shows the percentage of resection errors (mm) in overall 3618 recordings. They were the difference between the actual measurements taken intraoperatively and the original surgical plan. 24% had resection error of 0.0 and 90% showed a resection error less than 1 mm.

Resection error	Percentage
0	24%
0.1–0.5	49%
0.6–1	17%
1.1–1.5	4%
1.6–2	4%
2.1–2.5	1%
>2.6	1%
Total	100%

**Table 2:** Resection errors in each of the Six Bone Cuts. The table represents the range of resection errors recorded starting from no error to more than 2.6 mm, in each of the different bone resections recorded, namely Distal Femur Medial Condyle, Distal Femur Lateral Condyle, Posterior Condyle Medial, Posterior Condyle Lateral, Tibial Medial Condyle, Tibial Lateral Condyle.

Resection error	DFC Medial	DFC Lateral	Post Condyle Medial	Post Condyle Lateral	Tibia Condyle Medial	Tibia Condyle Lateral	Percentage
0	89	39	27	17	78	38	24%
0.1–0.5	71	112	114	110	70	114	49%
0.6–1	27	32	32	44	40	32	17%
1.1–1.5	5	9	10	14	4	9	4%
1.6–2	9	7	7	9	8	5	4%
2.1–2.5	–	2	6	3	–	1	1%
>2.6	–	–	5	4	1	2	1%
Total	201	201	201	201	201	201	100%

**Table 3:** Mean errors of different bone resections. The bar graph represents the mean error recorded in each of the different bone resections recorded, namely Distal Femur Medial Condyle, Distal Femur Lateral Condyle, Posterior Condyle Medial, Posterior Condyle Lateral, Tibial Medial Condyle, Tibial Lateral Condyle. The lowest error was recorded in Distal Femur Medial Condyle at 0.38 mm and highest was in Posterior Condyle Lateral at 0.60 mm.

Type of resection	Mean error (mm)
DFC Medial	0.38
DFC Lateral	0.41
Post Condyle Medial	0.55
Post Condyle Lateral	0.60
Tibia Medial Condyle	0.44
Tibia Lateral Condyle	0.44
Overall average of all resections	0.47

Postoperative radiographic analysis revealed good correlation with pre-operative CT planning guidelines. At discharge, all 201 knees achieved more than 100 degrees of knee flexion within 6–10 h after surgery with 89.5% reaching 120° by the 4–6-week mark at follow-up.

Postoperatively, one patient aged 86 had a dislocation post fall after being mobilized whilst the spinal was still wearing off after surgery. Poor soft tissues also contributed to this. A liner change with a 4 mm increment with hinge knee bracing for 4 weeks resulted in a stable knee. The patient had a stable knee with 0–110° at the most recent follow up of 22 months. No components were revised in this series to date, at a mean follow up of 17.4 months (range 7.1–23.8 months). Two other patients had postoperative stiffness requiring manipulation under anaesthesia at 6 weeks due to swelling after inadvertent over anticoagulation (given daily prophylactic enoxaparin 40 mg subcutaneous and oral aspirin 300 mg) resulting in local haematoma and reduced flexion. Our usual chemoprophylactic protocol is aspirin oral 300 mg daily for 6 weeks. The haematomas resolved without surgical drainage with improved range of motion.

## Discussion

PSI, a new advancement in TKA, is reported to be cost-effective compared to conventional techniques, with reduced hospital stay and low sterilization costs [16]. Stirling et al. [17] stated there wasn't enough published data to convincingly conclude in favour of CT or MRI for accuracy of pre-operative imaging in PSI. However, we used CT Scans for our patients as it was readily available with easy access to patients, cost effective, relatively safe and had a longer production to surgery shelf-life than MRI as cartilage surfaces change faster than bony surfaces. Thus, using MRI could potentially reduce accuracy of 3D block placement. Some of our patients were from the countryside and elderly with pacemakers (Mean age 67.72 years) where MRI was not suitable.

Our study appears to be the largest study to date in literature evaluating the accuracy of 3D cutting blocks and is aimed at determining the accuracy of the actual intra-operative resections vs proposed resections. Caliper readings post resection on comparison with the proposed resections, revealed that 90% of the overall readings showed resection error ≤1 mm (*P* < 0.0001). The mean error of different resections was recoded ≤0.60 mm. The highest error was 6.7 mm for the posterior lateral condyle, which was due to an error in the four in one resection jig where the sliding mechanism was jammed, taking a larger than measured posterior condylar resection. This was unfortunately noted after the cuts were performed in that case and a 14 mm poly liner was used instead of the planned 10 mm liner. All subsequent jigs were checked for this before cuts were made without any further issues. The computer assisted manufacturing technology had taken into consideration the presence of the peri-articular osteophytes in the preoperative templates. These were convenient for the placement of PSI blocks around them as the CT plans incorporated the osteophytes. All 201 knees achieved more than 100 degrees range of motion within 6–10 h after surgery with our enhanced recovery program [14] and had satisfactory clinical and radiological outcome. No intraoperative complications were noted from the patient-specific TKA with an exceptional 0% infection rate till the most recent follow up at 23.8 months.

A study conducted by Lustig et al. [18] that recorded resections of 45 patients found that the patient specific cutting blocks were within ±2 mm of the plan for 87.7% of the sample for both femoral cuts. The mean differences between the plan and the measured medial and lateral tibial resections were not significantly different to zero. Another study conducted by Yeo et al. [19], that recorded resections in 26 patients found that 85% of all collected readings were below the error margin of ≤1.5 mm, and 75% of the total readings were ≤1 mm more than the original template. There were only 7% of the overall readings that were ≥2.5 mm more than the original preoperative template. In 12% of measurements, there was no error or deviation from the templated resection (0.0 mm). Our study while still correlated with both, offers even better results. We recorded 3618 measurements (average 1206 readings) from 201 patients and found that 94% of collected measurements were below the error margin of ≤1.5 mm and 90% were below the error margin of ≤1 mm. In our study, 24% showed that there were no errors or deviation from the templated resection (0.0 mm). This may be due to a combination of enhanced 3D PSI manufacturing technology with improved surgical plans with specific markings to ensure accurate positioning of blocks.

More recently, robotic assisted TKA is gaining popularity, but there isn't enough evidence to state that it is superior to navigated, instrumented or PSI TKA. Liow et al. [20] illustrated that early experiences with robot assisted TKA, found the postoperative coronal mechanical alignment to be within 3°, with a mean alignment of −0.4 ± 1.7°, confirming the accuracy of the preoperative surgical plan, bone cuts and yielded 100% implant sizing accuracy [21].

There are limitations to this study. There may be elements of measurement bias, as the operating surgeon viewed the plans just before surgery and the same surgeon measured the resections. This was done to maintain consistency in measurements. Further, 3 separate measurements in 3 different planes were taken and averaged, then recorded independently. We did not perform a detailed radiographic analysis post-operatively. However, no components were revised for mal-positioning. Ligament balancing cannot be predicted preoperatively through CT scan, but with careful soft tissue releases, the surgeon was able to consistently keep to the surgical plan achieving good patella tracking and ligament balancing and stability. In conclusion, the 3D printed cutting blocks with slots for jigs accurately predict bone resections in PSI total knee arthroplasty which would directly affect component positioning.

## Conflict of interest

The authors declare that they have no conflict of interests.
